# Exploring the potential consequences of the disposable vape ban in the UK: A qualitative study with young adults who use disposable vapes

**DOI:** 10.1371/journal.pgph.0004686

**Published:** 2026-03-11

**Authors:** Richie J. Carr, Sara W. Alattar, Lana A. Al-Rifai, Hazel Morfett, Jasmine Khouja

**Affiliations:** 1 School of Psychological Science, University of Bristol, Bristol, United Kingdom; 2 MRC Integrative Epidemiology Unit at the University of Bristol, Bristol, United Kingdom; Muhlenberg College, UNITED STATES OF AMERICA

## Abstract

When the United Kingdom government announced a disposable vapes ban from the 1^st^ June 2025 in response to a rise in youth vaping, it was not clear how it would impact adults in the United Kingdom who used disposable vapes. In this qualitative study, we recruited and interviewed 22 young adults (n = 20 aged 18–22 years, n = 2 aged 29–30 years) residing in the United Kingdom who regularly used disposable vapes and self-reported either: having never smoked cigarettes, having previously smoked, or currently smoking. In online semi-structured interviews, we explored participants’ experiences of vaping and smoking, what they might do once disposable vapes were banned, and how they thought the ban would impact other people. The data was analysed using reflexive thematic analysis. Three themes were constructed relating to our research aims: “reasons for using disposable vapes”, “personal impacts of the ban”, and “wider impacts of the ban”. Most of our sample were female psychology students recruited via the University of Bristol. Recruitment primarily occurred in and around the University of Bristol campus and via the website. Participants were largely supportive of the ban due to the rise in disposable vape use among young people. Many young adults said they would switch to reusable/rechargeable vapes after the ban while others said they would stop vaping. Some participants (including some who had never regularly smoked before) said they might/would smoke cigarettes instead. Some suggested the ban would reduce current illegal sales of disposable vapes and others thought it may increase them. This study suggests the ban could have intended and unintended consequences for young adults who use disposable vapes, including increasing the use of cigarettes among some of these individuals. The findings highlight areas for future observational studies investigating the impacts of the ban and could aid identification of mitigating factors of unintended consequences.

## Introduction

Electronic cigarettes (e-cigarettes or vapes) are battery-powered devices that heat a liquid, often containing nicotine, for inhalation, referred to as ‘vaping’ [1]. E-cigarettes can aid smoking cessation and are generally considered less harmful than combustible cigarettes as they contain fewer harmful chemicals and toxicants, but they are not risk-free [2–4]. E-cigarettes can be reusable or disposable; reusable vapes (reusables) are refillable, rechargeable e-cigarettes [5] whereas disposable vapes (disposables) are single-use, pre-filled with e-liquid, and discarded after depletion [6]. Disposable vapes gained popularity due to their affordability, ease of use, and lack of maintenance requirements [7]. Modern disposable vapes feature sleek designs, a variety of colours and flavours, and branding strategies that may appeal particularly to younger demographics [8]. However, concerns emerged regarding the appeal of disposable vapes to young people and those who have never smoked, whose health could be negatively impacted by vaping; as highlighted by an evidence review, biomarkers found in people who vape but have never smoked indicate that vaping may increase their risk of respiratory and cardiovascular issues [1]. Aside from the direct health effects of vaping, vaping may also impact smoking behaviour. A study using data from the Millenium Cohort Study has reported that among children who have never smoked, e-cigarette use by age 14 is associated with an increased odds of tobacco smoking by age 17 [9]. Systematic reviews have also reported that young people (aged ≤30 years) who vape appear to be more likely to go on to smoke cigarettes, although it is unclear whether e-cigarette use is the cause of subsequent cigarette use or if there is a shared liability between the two behaviours [10–12]. Furthermore, Begh and colleagues found weak evidence of a negative relationship between vaping and smoking at the population level, indicating that e-cigarettes could be displacing cigarettes to a degree. Therefore, reducing the availability or appeal of e-cigarettes in response to the increase in popularity could impact vaping and smoking rates among the population.

In the UK, calls to reduce the appeal and availability of vapes to young people increased when the introduction of new disposable vape models in 2021 coincided with a rise in vaping prevalence, particularly among youth in Great Britain. The percentage of 11–17-year-olds who have ever vaped rose from 11% in 2021 to 20% in 2023. The percentage did not significantly change between 2023 and 2024 (18%), and current vaping rates among this age group remained at 7.2% [13]. The proportion of youth reporting the use of disposable vapes as their main device over this time period rose from 7.7% in 2021, to 69% in 2023, and remained relatively high at 54% in 2024 [13]. While these trends suggest the rise in youth vaping is stabilising [14], concerns persist regarding the appeal of disposable vapes and the long-term effects of vaping among youth.

In response to the rise in youth vaping, the UK government announced a plan in January 2024 to ban the sale of disposable vapes from 1^st^ June 2025. Prior to 1^st^ June 2025 disposable vapes were widely available in the UK in local convenience shops (aka corner shops/ off-licences), supermarkets, and other stores such as phone shops. After the ban, it became illegal to sell or supply a disposable vape in the UK, but reusable vapes that can be recharged and refilled continued to be legal to sell. Before the ban was announced, reusable vapes were also available, but refills were mainly available in specialist vape shops and online, with limited availability in supermarkets [15].

Given the popularity of disposable vapes among youth, the ban could effectively discourage youth vaping or encourage use of more sustainable vaping products. However, this policy may have unintended consequences, particularly for the 2.7 million adults who used to smoke cigarettes and rely on vaping as a harm reduction tool. Among them, 28% use vapes to aid smoking cessation, while 21% rely on them to prevent relapse [16]. Removing disposable vapes, could have limited accessibility of vapes and risked relapse to smoking for adults who were trying to stop smoking. At the time of the ban announcement, reusable products were more expensive and refill options were less accessible than disposables and cigarettes. As a result, if a reusable ran out of e-liquid, a person may have been faced with the choice of experiencing withdrawal, purchasing a costly new starter pack, or opting for the cheaper but more harmful option of buying a pack of cigarettes [17]. The ban on disposable vapes could have also significantly affected those who have never smoked. Previous research found that the age group most impacted would be those aged 18–24, with 7% of people who have never smoked in this group currently using disposable vapes, compared to 1.5% in the general population [18]. It was uncertain how those who have never smoked but were addicted to nicotine would react if they lost access to their usual product, as some may have quit while others might have sought alternatives. This could have led to the use of more harmful nicotine products, potentially increasing health risks rather than reducing them. Action on Smoking and Health (ASH) indicates that among people younger than 18 years who vape, 26% reported strong, very strong or extremely strong urges to vape in 2020, compared with nearly half (47%) in 2025 [14]. Given the increase in vaping among this age group over this time was likely due to disposable use among people who do not smoke [19], it is likely that a substantial proportion of young adult non-smoking nicotine users are addicted. However, this assumption is not yet supported by evidence.

The ban could also have affected up to 1.2 million adults who currently smoked, some of whom may have been young adults who also vaped, potentially disrupting their harm reduction strategies [18]. Research has suggested that product diversity and the ability to personalise vaping experiences are critical factors in user satisfaction [20]. Restricting consumer choice by banning disposables may therefore impact the effectiveness of vaping as a smoking cessation tool, ultimately impacting broader public health objectives.

Prior to the ban, there were also concerns that restricting access to disposable vapes while they were in high demand could lead to the emergence of illicit markets, as seen in Finland, where legislation introduced in 2016 banned the sale of flavoured e-liquids other than tobacco flavour. Despite this restriction, 43% of e-cigarette users surveyed between 2014 and 2018 reported continued use of non-tobacco-flavoured e-liquids, indicating that access persisted through alternative, and potentially illegal, sources [21]. Furthermore, bans on specific vaping products have historically led to unintended shifts in product innovation and demand. After the U.S. Food and Drug Administration (FDA) banned flavoured cartridges, online searches for Puff Bars — a disposable vape product — rose sharply [22]. A year later, vaping prevalence among individuals under 21 had not decreased, and disposables became the most commonly used device among both adolescents and adults [23,24]. This suggests that rather than reducing overall vaping, the FDA ban may have inadvertently driven users toward alternative products, potentially accelerating the rise of disposable vape use among young people.

There are also many other factors that contribute to the appeal and use of e-cigarettes outside the product itself. Authors of a qualitative study of young people (aged 16–20 years) who used disposable vapes have found that disposable vaping is seen as a social activity that they used to foster belonging and ‘fit in’ [25]. If social factors such as these influence use, then they may also have impacted anticipated and actual responses to the ban (e.g., young adults’ intentions and behaviour may have been influenced by peer group decisions). While the ban aimed to reduce youth vaping, understanding the anticipated impact on those who used disposable vapes (including those who used to smoke, those who have never smoked, and those who currently smoked when the ban was announced) is important to guide future observational research on the actual impact. Furthermore, gaining a deep understanding of how and why people who use disposable vapes anticipate changes in their behaviour in response to restrictions could aid public health messaging to mitigate unintended consequences. Currently, there is no qualitative evidence on how adults who used disposable vapes in the UK felt about the ban prior to implementation or how they thought their behaviour would change in response to the ban. Therefore, this study aimed to qualitatively explore the potential consequences of the ban of disposable vapes in the UK, focusing on its impact on young adults who use these products.

## Method

### Ethics statement

This study was reviewed and approved by the School of Psychological Science Student Research Ethics Committee at the University of Bristol (reference: 16282). Written informed consent was obtained from participants via a digitally signed consent form.

### Design

This study followed the methods outlined in our pre-registered protocol (https://doi.org/10.17605/OSF.IO/QEZ4A). A qualitative design was used with one-to-one online semi-structured interviews. The interviews explored participants’ patterns of disposable vape use and their opinions on how the ban on disposable vapes would impact them and other adults who use disposable vapes.

### Participants

We anticipated that a sample size within the range of 18–36 would be sufficient to reach adequate information power, based on previous qualitative studies that had a similar scope to ours [26–29]. During recruitment we did not balance gender as we did not anticipate any major gender differences and did not aim to investigate gender differences. This approach is in line with previous similar studies [25]. We recruited participants who self-reported that their vaping and smoking behaviour aligned with one of the following descriptions:

1. Never smoked cigarettes and uses disposable vapes five or more times a day for at least three months.
2. Smokes at least five cigarettes per day and uses disposable vapes five or more times a day both for at least three months.
3. Used to smoke five or more cigarettes a day for at least three months, attempted to stop in the past year, and then replaced smoking with disposable vape use for at least one month.

The definitions were based on previous work by Khouja and colleagues [30]. We recruited participants who self-identified with these groups with the aim of capturing a diverse sample of young adults with respect to smoking history, not to investigate differences between groups.

Recruitment took place in 2024 between the 22^nd^ of February and the 30^th^ of September, after the announcement of the ban but prior to implementation. We recruited participants who were living in the UK, aged 18–30 years old, fluent in English, able to give informed consent, able to attend an online session using Zoom, and comfortable discussing addiction. Participants could not take part if they had uncorrected vision or hearing problems. We focused on this age range for three reasons: First, the Smoking Toolkit Study and ASH report that the highest prevalence of vaping is among young adults aged 16–24 and 25–34 respectively and ASH also report that among adults, those aged 18–24 are the age group with the highest proportion using mainly disposable devices in 2025 [31,32]. Second, we felt that there may be a generational difference between those aged 30 years and or less and those aged more than 30 years given that e-cigarettes were not readily available in their youth (a 31-year-old would have been 20 years old in 2013 when e-cigarettes were becoming popular in the UK). Third, it is illegal to sell e-cigarettes or cigarettes to anyone below the age of 18 in the UK, so we did not include those under 18 years of age in the study as we anticipated their opinions and experiences could substantially differ from those who can legally purchase nicotine products such as vapes and cigarettes. Therefore, studying the age group 18–30 is an age group that should be more representative of people who legally used disposable vapes prior to the ban.

Participants were recruited via the University of Bristol’s experimental hours scheme (which reimburses students with course credits for their participation in research), posters around the University of Bristol campus and surrounding area in Bristol, an advert in the Tobacco and Alcohol Research Group newsletter and website, and word of mouth. Participants recruited through the University of Bristol’s experimental hours scheme received 0.75 credits; participants recruited through any other method received a £10 Love2Shop voucher.

### Materials and measures

Participants provided informed consent via a digital Qualtrics form, agreeing to the terms of the study (http://www.qualtrics.com/). Participant characteristics and information (age, gender, ethnicity, duration of regular vape use, vape brands used, and nicotine strengths used) were also collected using a Qualtrics survey (S1 Table).

An interview schedule was developed before the interviews that included open-ended questions with prompts to keep the focus on the research aims while allowing the participant to elaborate on questions and give in-depth answers. The schedule was an adapted version of the topic guide used by Khouja and colleagues, who investigated the potential impacts of an e-liquid flavour ban – a qualitative study that was similar to the present study [29]. The schedule aimed to elucidate the participants’ patterns of vape use, what they would do once disposable vapes were banned, and how the ban might impact other people. Questions were consistent across the three participant groups, except for questions that were dependent on smoking status (see S2 Table, S3 Table, and S4 Table for the full interview schedules).

Interviews were conducted (and recorded) using a virtual meeting platform: Zoom (version 5.17.11) and data were analysed using qualitative data analysis software: NVivo (version 20.7.2).

### Procedure

Interviews were expected to last approximately 20 minutes and were conducted by three research assistants (RAs), each interviewing one group of participants. At the time of data collection, all RAs were students at the University of Bristol and living in Bristol. RA1 used to smoke cigarettes regularly and occasionally used both refillable and disposable vapes. Both RA2 and RA3 have no history of smoking and vaping. RA1 interviewed those who self-identified as having never smoked, RA2 interviewed those who self-identified as having previously smoked, and RA3 interviewed those who both smoked and used disposables. The participant was sent and instructed to read an information sheet before joining the Zoom call on the day of their interview. The researcher began by reminding the participant of the study procedure and of their right to withdraw from the study. The participant was then given another opportunity to read the information sheet and asked to sign the digital consent form via a hyperlink sent by the researcher over Zoom. By electronically signing the consent form the participants also confirmed they fit the study inclusion criteria and that they were eligible to take part in the study. Once their responses were registered, they completed the Qualtrics survey (again via hyperlink). The participant was reminded that there were no right or wrong answers to the interview questions and the interview (and recording) began, following the topic guide. The interviewers aimed to keep any questions or prompts that deviated from the interview schedule open-ended so as not to lead the participant to answer in a particular direction. Once the interview was complete, the recording was stopped, and the researcher sent an email to the participant containing a debrief sheet and, if recruited outside of University of Bristol’s experimental hours scheme, the code to redeem their reimbursement voucher. The audio recordings were transcribed verbatim by hand by RA1 after the interviews and any information that could be used to identify the participants were removed or changed (for example the name of a local pub). The transcripts are available online at the University of Bristol data repository, data.bris, at https://doi.org/10.5523/bris.3n5i9qcm8p50f2r6leehw0rns0 [33]. The transcripts were not returned to participants for comment or correction.

### Data analysis

The study was designed to gain insight into what might happen to people who use disposable vapes after the ban, based on their opinions and experiences, thus capturing patterns of meaning across the dataset, rather than focussing on unique experiences. Therefore, we analysed the data using the reflexive thematic analysis (RTA) approach [34]. We approached the analysis using constructionist assumptions, acknowledging that there are social factors and systems that contribute to the construction of participants’ experiences and meaning making. We adopted an experiential (rather than critical) orientation to data analysis, as our focus was to explore the experiences and opinions of the participants, not to interrogate the social factors and systems that may have contributed to their construction [35,36].

Data were analysed following the six stages outlined by Braun & Clarke [37]: data familiarisation, coding, generating initial themes, developing and reviewing themes, refining and naming themes, and writing up. In the early stages, RA1 immersed himself in the data by re-reading the transcripts multiple times and making notes. Both semantic and latent codes (conceptualised as a single unit of meaning) were developed using an inductive (bottom-up) approach, focussing on the participants’ perspectives. There will have been *aspects* of deductive (top-down) coding but only given RA1’s background experiences and knowledge of the topic – coding was not conducted to prove a theory or match pre-determined codes. Any codes that communicated the same meaning were combined, and initial themes and sub-themes were developed by combining codes that were conceptually similar. Distinct themes (and sub-themes) were developed that captured different aspects of the dataset. The importance of a theme for inclusion in the results was not recognised solely by the recurrence of information, but by its meaning in relation to the research aims.

The data was only analysed by RA1. Some qualitative approaches use multiple-coders and inter-rater reliability to analyse data, however this assumes an accurate reality in the data that can be captured through coding [38]. In RTA, the researcher inevitably brings their interpretations and assumptions to data analysis as coding is an *active* process - RTA is more concerned with authenticity and openness rather than objectivity and reliability [39]. RA1 reflected on and challenged his assumptions and interpretations during data analysis, and outlined his positioning during the research, aiming to make any potential influences on data analysis open (S1 Text). RA1 also met with the other authors to discuss the themes he had constructed with the aim of deepening interpretation and surfacing assumptions (not to analyse the data via inter-rater reliability).

## Results

We recruited and interviewed 27 participants in total. During the interviews, most of the participants who self-reported having never smoked said they had tried a cigarette in the past, but they still self-identified as people who had never smoked. Never smoking is often interpreted as having never regularly smoked cigarettes [40,41]. Thus, these participants were included in the analysis. Similarly, one person who self-identified as previously smoking cigarettes said they occasionally smoke in social settings; this still fits our criteria, so this person was also included in the analysis. However, five participants shared other information about themselves which highlighted that they did not meet the eligibility criteria that they had previously confirmed they met. These interviews were excluded from analysis. Details of why these participants were excluded are in S2 Text. Twenty-two participants were included in the final analysis and self-identified as: having never smoked (n = 9), having previously smoked (n = 7), and currently smoking (n = 6). No further interviews were conducted as there was enough data in the 22 interviews for a rich exploration of our aims. Some participants seemed eager to discuss the subject of disposable vapes and talked about the subject at length, while others were more withdrawn during the interviews and provided less detailed answers to questions. The length of the interviews included in the analysis ranged from 11 to 41 minutes.

Table 1 shows the participant characteristics of the included participants by group. The majority of participants were white, female, and in the lower range of 18–30 years old. Half of the participants reported using disposable vapes for 3–6 months, and the most commonly used disposable vapes were ElfBar and Lost Mary. Two participants used nicotine strengths over the UK legal limit (20 mg/ml). At least 12 participants were university students (recruited via the experimental hours scheme meaning they were psychology students at the University of Bristol), and likely more, as the remaining participants were recruited via adverts on campus, in the surrounding area, and online.

**Table 1:** Participant characteristics.

Category and response options^a^	People who self-identified as having never smoked (n = 9)	People who self-identified as having previously smoked (n = 7)	People who self-identified as currently smoking (n = 6)	Total (n = 22)
Gender				
Female	8	4	4	16
Male	1	3	2	6
Ethnicity				
Arab	0	0	3	3
Asian/British	1	0	0	1
Mixed/Multiple ethnicities	4	0	0	4
Turkish	0	0	1	1
White	4	7	2	13
Age (years)				
18	2	0	1	3
19	3	2	0	5
20	2	3	2	7
21	0	1	2	3
22	0	1	1	2
29	1	0	0	1
30	1	0	0	1
Duration of regular vape use				
3-6 months	3	5	3	11
6-12 months	1	0	0	1
1-2 years	4	1	1	6
2-3 years	0	1	1	2
3-4 years	0	0	1	1
4-5 years	1	0	0	1
Brand/s of disposable vape used				
Crystal	4	2	1	7
ElfBar	5	4	3	12
Elux	0	1	0	1
“Fake products from China”	0	0	1	1
Fumot	0	0	1	1
Hayati	0	2	0	2
JUUL	0	0	1	1
Lost Mary	4	5	2	11
Mazaj	0	0	1	1
RandM	2	1	0	3
“Various 9000 puff stuff”	1	0	0	1
Vuse	0	1	0	1
Nicotine strength/s used				
20mg/ml	8	7	5	20
30mg/ml	1	0	0	1
50mg/ml	0	0	1	1
Recruitment method Ex. hours Non ex. hours	6 3	5 2	1 5	12 10

^a^Response options are excluded where no responses were recorded.

Five themes were constructed in the analysis (Table 2): “reasons for using disposable vapes”, “personal impacts of the ban”, “wider impacts of the ban”, “thoughts on cigarette and vape products”, and “reasons for vaping generally”. As they provide context but are not relevant to the research aims, “thoughts on cigarette and vape products” and “reasons for vaping generally” are included in S3 Text. Here we represent each participant with a letter followed by a number: the letter indicates the participant’s group (‘N’ = participants who self-reported as never having smoked ‘D’ = participants who reported dual use of cigarettes and vapes, ‘P’ = participants who reported previously smoking cigarettes) and the number indicates their order in the interview process for that group (e.g., N02 was the second person interviewed who self-identified as having never smoked).

**Table 2:** Themes and sub-themes.

Themes	Sub-themes
Reasons for using disposable vapes	Ease and accessibility Social influence Psychological differences in the use of disposable vapes Taste
Personal impacts of the ban	Vaping intentions Intentions to use cigarettes or nicotine alternatives Perceptions of vaping
Wider impacts of the ban	Illegal market Switching to other vapes Smoking
Thoughts on cigarettes and vape products	Comparison of smoking and vaping Disposable vapes compared to reusable vapes
Reasons for vaping generally	Habit and addiction Ease of vaping Health reasons Social influence

### Theme 1. Reasons for using disposable vapes

The theme “reasons for using disposable vapes” includes participants’ reasons for using disposable vapes specifically (not general reasons for vaping such as addiction). This includes initial as well as current reasons for using disposables, and why participants use them over reusables. Four sub-themes were included: “ease and accessibility”, “social influence”, “psychological differences in the use of disposable vapes”, and “taste”.

#### Ease and accessibility.

Many participants said they used disposable vapes because of their widespread availability. When asked what attracted them to disposable vapes, D01 said: *“the accessibility primarily, like every shop sells them.”* N02, who had tried a cigarette in the past, said they had previously used a reusable pod (cartridge) vape – a rechargeable device with replaceable pre-filled pods that slot into the device. N02 described how it was more cost effective than disposable vapes: “*you can get one pack of pods for… five pounds I think, or six pounds or something like that but it had two pods in that lasted the same amount of time as a singular… disposable vape.”* However, they said they used disposables over reusables due to their accessibility, noting that: “*the disposables you can obviously buy in like every… corner shop Tesco Sainsbury’s like everywhere, whereas the pods you normally have to go to a actual vape shop to get, which is obviously quite inconvenient.”* Other participants also noted that reusables are less easy to use due to the maintenance required, and that this influenced them to use disposables. For example, P01 said: *“it just becomes a lot easier to just get a disposable vape rather than… erm carrying around a device and then getting the refillable parts for it, erm so convenience plays a role erm in that.”*

Some participants also stated that the ease and accessibility of disposable vapes meant they were more appealing when they were underage, as N01 (who had tried a cigarette in the past) described: *“when I was below 18, it was way easier to just go and buy erm… a disposable, because like corner shops would sell them like to underage children, erm whereas the vape shops that sold the reusables, they wouldn’t do that.”* P03 said: *“my parents didn’t let me vape or smoke, so, I got a disposable one so I wouldn’t regularly have one at my house.”* D05 described the ease of using a disposable vape in school compared to smoking. When discussing what initially attracted them to disposable vapes, they said: “*you can just buy one vape and smoke [vape] in the bathroom and you don’t need to go outside [...] it’s easy.”*

#### Social influence.

Many participants described how their disposable vape use was influenced by other people. D01 described their first use of a disposable vape as *“hopping on the trend.”* P04 said: *“I only picked it up when I was here [at university] because a lot of people in my flat do.”* Similarly, D02 said: *“it was just erm quite attractive at the time because all your friends and your peers do it, so yeah, I guess through friends.”* Some participants also said that initially they felt cool using disposable vapes. N05, who had tried cigarettes a few times, stated: “*I think because like when I was fifteen, I thought it was cool, sadly enough. That’s pretty lame but at the time I thought it was like this new thing.”*

Some participants said their disposable vape use started by trying other peoples’ on ‘nights out’ (evenings spent outside of the home, usually in bars or public houses). For example, N06, who would occasionally share a cigarette with a friend, said: “w*hen we were going on a night out my friends would have them and so I’d try theirs [...] so I’d be like ‘ok I should get my own now so that I can share it with them’.”* Other participants described similar scenarios at festivals. N02 said: *“I was at a festival, and… everyone was… everyone had one [a disposable] and I had never like, heard of them before, erm… so then I tried one, and then… I ended up buying one, and then… just went from there.”* Similarly, N04 (who had tried a cigarette in the past) said: *“I remember it was like £5 which at the time I thought was… really cheap, so I was like yeah why not, erm… so yeah, I bought it from her [a friend] at a festival. And that’s where it started.”*

#### Psychological differences in the use of disposable vapes.

Throughout many interviews, reusables were described as ‘proper’ or ‘actual’ vapes, and some participants said they used disposables (rather than reusables) because buying a reusable vape felt like a commitment to vaping, whereas using a disposable vape made them feel like they were not vaping as frequently as they were. When asked if they had ever tried a reusable vape, P04 said: *“I think because I keep telling myself I’m going to quit, that I can’t bring myself to buy one of the actual ones [laughs].”* Similarly, D02 said: *“part of it is if I feel [if] I’m getting a reusable vape, that’s really accepting that I have an addiction, and dependency of some sort.”* N06 stated: *“it feels like a bigger commitment [buying a reusable] and I don’t want to admit it to myself that I vape as much as I do which maybe is why… another reason why I’ve not… got a refillable one yeah.”*

Some participants also described how there was an attraction to disposable vapes because of their cheap prices compared to reusables, but that this was deceiving. For example, when asked why they used disposables over reusables, N07 (who had never tried a cigarette) said: *“even though it probably adds up, that I am probably spending more or at least an equivalent amount, it just seems like a… like a lower mental barrier that it’s a... it’s a low price.”* D08 similarly described how they initially thought that a cheap single use vape would be a short-term investment, but that this was not the case:

“*The short term aspect of it and everything made you, made me think at least, that I’m not committing to a vape machine that maybe costs three times the price of a normal vape [...] I thought to myself I’ll just have one for enjoyment, and then get rid of it, but… they’re they turned out to be a lot worse than what a vape machine kind of… other forms of nicotine intake are… now it’s worse than that basically.”*

#### Taste.

Some participants said they used disposables because they preferred the taste compared to reusables. When asked whether there were any specific reasons they used disposable vapes, P03 said: *“they are nicer than the usual like… vape juices you get, erm…”* Similarly, D06 said: *“I’ve tried it [reusable vapes] before, erm… but I think the flavours in refillable pods are different, erm… so I didn’t continue using them.”* When asked what attracted them to disposable vapes, N01, noted that: *“the taste of… a disposable vape is so much nicer than cigarettes or… erm… reusable vapes I find.”* N01 did note that they once found an e-liquid (for refillable vapes) that they liked as much as disposables: “*[it’s] like ElfBar but ElfLiq, erm like liquid, erm… so it tastes very similar so, once I found that it was kind of like I had… a disposable, but it was way cheaper.”* N04 described how they did not like the taste of refillable tank-based vapes (reusable vapes with a reservoir that can be manually filled with separately purchased e-liquid): “*I found that the flavours of the… vape liquid weren’t quite as strong or as good as erm in the disposable vapes, so that kind of made me not want to use them as much.”* However, N04 also mentioned ElfBar as a brand that produced a reusable pod-style vape that tasted as good as disposable vapes, noting that: *“the flavour’s better and I think… erm still cheaper than just regular disposable vapes.”*

### Theme 2. Personal impacts of the ban

The theme “personal impacts of the ban” includes discussions around how the ban will impact the participants personally, including impacts on vaping and smoking behaviour, and how the ban has impacted their perceptions of vaping. Three sub-themes were included: “vaping intentions”, “intentions to use cigarettes or nicotine alternatives”, and “perceptions of vaping”.

#### Vaping intentions.

Despite preferences for disposable vapes over reusable vapes, most participants appeared unbothered by the prospect of switching to reusable vapes and some stated their intention to make this switch. P01 said: *“I think I would just swich to refillable vapes, erm… yeah I think they’re… they’re a close substitute, so… erm I don’t think I would… have much trouble erm in making that switch, I think it would be quite smooth.”* P02 said: *“Erm [I’ll] probably just switch to a… like a non-disposable one, you know the refillable ones erm… yeah I guess I’ll do that.”* For other participants, their intentions to use other vapes after the ban appeared to depend on what other people will do. N06 initially said the ban would be good motivation for them to stop vaping: *“I think if I don’t have like access to them all the time… it… will be like a good incentive for me to stop altogether.”* However, they also described how their friends might influence them to buy reusables: “*I feel like considering the first time I vaped I only wanted to vape my friends’ vapes I didn’t want to become a vaper, I think… realistically if they ban disposable vapes I probably would end up buying refillable ones.”* P03 described how they will probably buy a reusable vape, due to being at university: *“Erm… yeah [I’ll] just-just buy a normal vape [...] it just feels like a thing that’s constantly around us and it’s quite difficult to quit something that’s constantly there.”* N04 stated that they would not like to switch to reusable vapes, noting that: “*I think they’re not as big of a thing [compared to disposable vapes].”* When asked to elaborate, they said: *“Erm… like that’s what initially got me onto disposable vapes is-is seeing everyone and wanting to try it out [...] I think people who have stopped using disposable vapes have gone to… erm smoking not to reusable… vapes, erm from just the people around me and so… yeah.”*

A few participants said they would probably stop vaping after the ban. When asked what they might do after the ban, N07 said: *“[I’ll] probably use it as an opportunity to stop vaping.”* N10 said: *“do you know when you’re like ok I’ve got this addiction that I didn’t mean to and I don’t know why, but it’s quite bad now [laughs] so I’m just going to try and be like ‘fine, that’s it’.”*

Participants’ awareness of reusable vape products varied. Some participants discussed specific vapes in detail, while others had little to no knowledge of other products. When asked if they had ever tried a reusable vape, N11 said: “*Erm I might have bummed it off someone [tried someone else’s] but I don’t think I’ve like—I’ve never bought it, because I didn’t really know they existed.”* N10, who had tried a cigarette in the past, said: *“Oh I don’t think you can get reusables? You don’t—there’s no reusable I’ve never heard of a reusable one so I didn’t even know that that existed so maybe lack of knowledge of them existing if they exist.”* Although they later acknowledged that they had seen ‘pen-style’ vapes.

Two participants said they will continue to source disposable vapes. When asked what they might do after the ban, D07 said: *“I’ll get vapes from my home country.”* D05 insinuated they would purchase disposables illegally, saying: *“I’m going to get stronger products for cheaper prices. I hope they will ban it so we can get better products from China. [Laughs].”*

#### Intentions to use cigarettes or nicotine alternatives.

One participant (P05) said they might relapse to smoking cigarettes after the ban: “*I wouldn’t have a reusable vape again because it made me feel so ill, I’d maybe start smoking again but just try and have less. I’d probably try and quit to begin with, but I doubt it would last very long.”* P05 said that they had not completely stopped smoking cigarettes, but occasionally smoked in social settings. Most participants who self-identified having previously smoked had tried one type of nicotine alternative (nicotine products other than cigarettes and vapes, e.g., nicotine gum). While they did not discuss using nicotine alternatives after the ban, those who had tried alternatives described a preference for vaping. P04 said: “*I did not like Snus at all”,* and P07 said: *“[nicotine gum was] just not as good [laughs] again, I feel like it was too much… nicotine, comparatively.”* Some noted that the nicotine alternatives did not replace the hand to mouth action of vaping. P08 said: *“I think for me a lot of the addiction is the actual hand action, like putting a vape to my mouth, so Snus just doesn’t—I don’t understand how that could replace it.”* P04 said: *“I also like… the like stimulation of having something to put in my mouth all the time [laughs].”*

The majority of participants who reported both smoking and vaping said they would smoke more after the ban. When asked how the ban will affect their smoking patterns, D06 said: *“Erm yeah, I’ll probably just erm use cigarettes, erm… instead of vapes.”* D02 said: “*yeah it [the ban] would one hundred percent increase my smoking tenfold.”* D01 also described how smoking cigarettes as a replacement for disposable vapes would be an effective way to stop vaping:

*“In a way I think smoking cigarettes is actually a really effective way to quit, erm… because they’re so gross and they smell so horrible and they make your clothes and your breath and your fingers smell. So I think I would yeah use—smoke cigarettes*.”

This quote from D01 seems to reflect the dominance of disposable vapes among young people — rather than considering using e-cigarettes as a less harmful alternative to cigarettes, this person describes cigarettes as a means to stop vaping.

D05 said the ban would not increase their smoking, although this appeared to be due to the way they currently used disposable vapes: *“[it’s] just there for the taste and something to have in the hand it’s just a distraction [...] if you’re really smoking cigarettes and you’re really enjoying nicotine then it [disposables] will not help you at all, it’s just a toy in your hand.”* Experiences of nicotine alternatives among those who smoked and vaped (dual used) varied, but most did not discuss intentions to use nicotine alternatives (other than cigarettes) after the ban, except D08: *“I might go back to being a full time… smoker, instead of vaping, erm… I might also… I-I like I said I think I’m going to see—to try and see alternatives to vaping like Snus or patches, erm to see how those work out.”* Some participants who self-reported smoking and vaping also mentioned the hand to mouth action that is missing from alternatives:

D01: *“What was missing for me and partly what I think I am addicted to, is the actual feeling of something filling my lungs. Like I saw this TikTok where this guy said oh ‘it feels like a hug from the inside’. I was like, [whispers] ‘it does feel like a hug from the inside’. Like it feels warm and, you feel full and you don’t get that with, erm… those other alternatives, and I think that was kind of the downside of the patches and why I would still smoke two or three cigarettes a day when I was on the patches, was because, I missed that… you know.”*

Most participants who self-identified as having never smoked described elements of smoking they found off-putting and did not discuss smoking after the ban. However, some said they might smoke after the ban. N09, who had never tried a cigarette, said: *“I’d probably start smoking [after the ban], I’ve seen my flatmate does it a lot so I’d probably… I’d probably just do that.”* Similarly, N02 said: *“I’d probably start… smoking socially more [...] normally I don’t like… I don’t smoke really at all… but I’d probably… [pause] smoke… more.”* N11, who would occasionally smoke one cigarette, expressed possible intentions to smoke, although this was tentative. They noted: *“I don’t want to be like a smoker”* but later in the interview said: “*I don’t know I might become like a little bit more of a sociable smoker [after the ban] like I was before, but… I’m not sure.”* Most participants who self-identified as having never smoked had never tried any nicotine alternatives. One discussed using nicotine alternatives after the ban: while they said they would probably buy a reusable after the ban, N01 also said, *“I might just stay on Snus, to be honest.”* However, they noted that: *“it’s more expensive erm which means that I would use it less.”* N01 later clarified that they were referring to nicotine pouches, not Snus. Generally, it seemed there was a lack of understanding among participants of the distinction between Snus and nicotine pouches (i.e., whether the product contained tobacco or not).

#### Perceptions of vaping.

Many participants said their opinions on vaping and disposable vapes were unaffected by the ban because they were aware of a rise in (particularly youth) vaping, and thought it needed to be addressed. N05 said the ban had not impacted their opinions on vaping and that they supported the policy:

“*I started [vaping] when I was fifteen, that’s a perfect example of why it should be banned like, these kids like they market to kids, my friend had one that like lit up and like made all these colours, I was like, come on like, these are—they’re for kids like, it’s so obvious like, the companies can’t deny it, like… and I think the ban is less for people like me like adults who like can make their own decisions, and more for kids who are susceptible to marketing and stuff. I think it’s good.”*

D01 said: *“yeah, I’m surprised that it’s lasted this long, and I was very surprised when they started selling them in Sainsbury’s and Tesco’s and these like official supermarkets. It makes sense some off-licence, but that was it was kind of a, ‘really? Are you really going to normalise this in this way?’”* N06 said: *“disposable vapes are sold in like… pretty much every corner shop everywhere they’re so accessible [...] making it less available will like stop it from being so normalised and people will think about buying it more, erm, yeah.”* When asked whether the ban had impacted their opinions on vaping, P08 said: “e*rm… not massively [...] out of all policies, ever, I’m just like, this one makes so much sense, because… it’s not like you’re fully cutting people off [...] it’s just taking away that like candy flavoured stuff that kids now see as an after school snack.”* The responses of these participants show how they consider that disposable vapes have become normalised, particularly P08, whose depiction of disposables as an ‘after school snack’ for children, does not portray them as a harm reduction measure for people who smoke or used to smoke.

A minority of participants said the ban announcement had affected their opinions on disposable vapes. P04 described confusion around the government’s decision to only ban disposable vapes: *“I think the fact they’re considering banning them completely when they haven’t banned like… cigarettes and stuff, makes me do think that they [disposables]… they must be like… really, really bad for you.”* This highlights the impact that the impending restrictions had on this person’s perceptions of the relative harms of vaping and smoking.

### Theme 3. Wider impacts of the ban

The theme “wider impacts of the ban” includes participants’ opinions on how the ban will impact other people. This includes how it will impact the illegal market (including underage sales), and the impact of the ban on other peoples’ smoking and vaping behaviours. Three sub-themes were included: “illegal market”, “switching to other vapes”, and “smoking”.

#### Illegal market.

Some participants described how banning disposable vapes should reduce underage sales by removing them from shops that children currently have access to, off-licenses in particular. D06 said:

*“I’ve seen [in] off-licence younger kids erm buying vape without being asked for IDs, erm… right now it’s… it’s very much common, erm so them now [not] being able to kind of get someone older to buy it for them, or erm… kind of erm… just [not being able to] buy it from off-licence will probably erm make it less of an issue.”*

P03 described how the ban would reduce youth vaping. When asked how the ban would impact younger people who cannot legally buy vapes, they said: *“they’d stop, […] because the people that buy disposable vapes are often people who can’t go to like vape shops and buy proper ones, so they go to… corner stores that are… a bit… illegitimate.”* However, they also noted that children may switch to other cheap alternatives if they are available:

*“You know with all that kind of stuff like JUULs [a pod vape] were a thing and you can buy JUULs in a corner shop so they’ll probably go back to JUULs or… other cheap… alternatives that are… either disposable or so cheap that you can you know bin them if they don’t work anymore.”*

While some participants thought that removing disposable vapes from off-licences would reduce illegal sales, others argued that illegal sales may continue in off-licenses. P05 described how other banned products are currently sold, and that disposable vapes may be sold in the same way: *“I think it’s probably still going to be available in off-license shops, because they’re not… like 3500 puff vapes have been banned, but I have one right now, because there are a lot of shops that just like import them in.”* N01 said: *“I think shopkeepers will still continue to sell vapes, like under the counter, they’ll have them under the counter, I think that’s what they did erm with like erm… I don’t know if you remember like erm five percent vapes [...] the ones that are illegal now, but you can still get them in like certain shops, because erm… people still want them.”* Whether participants thought the ban would reduce or increase illegal sales of disposable vapes, corner shops and off-licenses were commonly discussed as a means of current or future illegal sales of nicotine products.

#### Switching to other vapes.

Some participants thought people who use disposable vapes will switch to reusable vapes after the ban. For example, D02 said: *“there’s so many people I know who got into vaping because of the fruity flavours of disposables. So, will, I take it they’ll just get a reusable, because they’re hooked now.”* Some participants made a distinction made between ‘casual vapers’ who might stop vaping and more frequent or ‘serious vapers’ who would switch to reusable vapes. P07 said: *“I think if they’re more serious vapers, they’ll probably just get a refill [reusable vape]. They’re not going to be happy about it obviously but if that’s the only alternative that’s what they’re going to have to do. I don’t think it will stop serious vapers, but it might stop… casual, social vapers.”* P02 said*: “the people who are like addicted and that vape on a daily basis will just switch to, you know, a refillable one but the people that vape less often, I’ve-I’d say for them maybe it [the ban] might deter them a bit.”* This depiction of the ‘casual social vaper’ stood out in the same way that was discussed in other sub-themes by participants. It similarly seems to reflect the apparent normalisation and growth in popularity of disposable vapes, as participants liken it to a casual activity that some people take part in, compared to ‘serious vapers’ who are more dependent on nicotine.

#### Smoking.

Some participants said that people who use disposable vapes might use cigarettes after the ban. D05 described how people who use vapes to smoke less cigarettes may return to smoking cigarettes: *“I think you’re really in a big problem if you’re really trying to stop smoking cigarettes and just like can’t buy vapes anymore, so they [will] just go back to smoking cigarettes.”* Some participants noted that they knew people who would switch to cigarettes after the ban. P04 said: “*I know a lot of people who like… smoke sometimes but usually have a vape, and so if the vapes got banned they would just start smoking all the time rather than just occasionally.”* P05 said: *“it [the ban] just doesn’t make any sense to me like I know a lot of my friends… who vape, they’re not going to be buying reusable vapes they’re going to start smoking, which is so much worse for you.”*

N11 described how people might replace the use of disposable vapes with cigarettes on ‘nights out’:

*“People get used to also like holding things as well whilst they like chat on nights out so like, if someone’s going to be smoking like and they can’t vape they might be like oh can I bum [have] a fag off you can I have like a cig, and then… you know… it will continue.”*

These assumptions regarding the actions of people they know highlighted a feeling that cigarettes may be the chosen option to replace disposable vapes for both people who are addicted to nicotine and those who only vape socially.

## Discussion

We aimed to understand how the UK ban on disposable vapes might affect young adults who use them, using the opinions and experiences they shared during interviews before the implementation of the ban. The results of our analysis include three themes that were relevant to our research aims: “reasons for using disposable vapes”, “personal impacts of the ban”, and “wider impacts of the ban”. These themes suggested the ban could have a variety of consequences for young adults, including impacting their vaping and smoking behaviour, and increasing illegal access to disposable vapes. Our results also highlight drivers of disposable vape use, including social influences, ease and accessibility, taste, and the perception of disposable vapes as different to ‘real’ or ‘proper’ reusable vapes. Most participants expressed strong support for the ban due to the rise in vaping caused by disposables. Participants appeared to distinguish ‘real’ (reusable) vapes from disposables and some described how their single-use made them feel they were not committing to vaping, despite using disposables regularly. For many, disposable vape use was also strongly influenced by other people and described as a trend. Despite preferences for disposables over reusables, many participants said they would switch to reusables after the ban, although some noted that whether they did so would depend on what their peers do. Other participants said they might stop vaping once the ban is enforced. Some participants expressed intentions to smoke as a replacement for disposables after the ban, and there was limited discussion of using nicotine alternatives as a replacement. Replacing disposable vapes with cigarettes was mainly discussed among participants who reported currently smoking and vaping. However, some who self-identified as having never smoked, and one who reported previously having smoked also discussed this. In addition, some participants suggested that other adults who use disposable vapes might smoke after the ban.

Although many participants in our study said they would switch to reusable vapes after the ban, some said they would use cigarettes instead. Thus, this study suggests that the ban could have caused some young adults to smoke, including those who self-identified as having never smoked cigarettes before the ban. However, quantitative studies should explore the extent to which this behaviour shift has occurred (or not) since the ban*.* If this shift has occurred, it would be concerning given the increased risk that cigarettes pose to health [3,42] and the proportion of never-smoking young adults who used disposable vapes before the ban [43]. Awareness of vape products other than disposables varied significantly, which may have affected intentions to use cigarettes, rather than other vaping products that will remain available, although some participants had a greater awareness of other products and still expressed intentions to smoke. Despite evidence suggesting half of adults in the UK believed that vaping is ‘at least or more harmful than smoking’ in 2024 [16], the majority of participants who said they might smoke appeared to have accurate perceptions of the relative harms of vapes compared to cigarettes, including participants who self-identified as having never smoked. This is somewhat in contrast to previous research finding that accurate perceptions of vaping (as less harmful than smoking) are associated with greater likelihood of switching from smoking to vaping among young adults who smoke [44], rather than the other way around. Intentions to smoke after the ban may be even greater among those who have *inaccurate* perceptions of the relative harms of vapes and cigarettes, but we are unable to infer this from our study [16]. Some participants in our study said they would stop vaping after the ban. Since the UK government announced their plan to ban disposable vapes, the increase in the number of people using vapes in recent years has stabilised. The number of people using disposables has declined, and there has been a shift towards reusable products [45]. Clear communication to young people and adults about the harms of vaping in relation to smoking is necessary to ensure that they do not take up smoking now. Unpublished data from a recent survey of local authorities in the UK (conducted by one of the authors) suggested that many have initiatives to educate young people, but not all are accurately communicating the relative harms or embedding their vaping education with smoking education. Given that some young adults will have been exposed to similar initiatives in their adolescence, more education targeting young adults may be needed.

Some participants discussed context-specific changes to their behaviour that they thought might occur after the ban. For example, some thought people might use cigarettes on ‘nights out’ to replace the hand-to-mouth action of vaping that had become habitual. Vaping and smoking both require hand-to-mouth movements, and research has reported this makes e-cigarettes useful for replicating cigarette smoking – vaping meets the psychological need to put something in the mouth [46]. Other qualitative studies with young adults who vape have also reported that the gestures and sensations of vaping become habitual [47]. Additionally, evidence has suggested that, among those who both smoke and vape, situational cues including alcohol use and the presence of other people who smoke are more greatly associated with cigarette craving (compared to e-cigarette craving) [48]. If young adults are no longer able to use disposable vapes in social settings like nightclubs and bars where tobacco is often used [49], the likelihood of them smoking cigarettes in these settings may increase, especially as many thought that reusable vapes were less accessible than disposables. However, the potential for increased cigarette smoking in night life settings was not discussed in-depth in this study and could be an area for future research to explore.

The participants indicated that their behaviour change in response to the disposable vape ban could depend on the extent to which they believed other products could replace their current product. For example, some participants described how they preferred the taste of disposables to reusables, but others described how they liked the taste of reusable ElfBar products as much as disposables and could see these as a potential replacement product. Disposable vapes contain nicotine in a salt form that is less harsh tasting compared to the free-base nicotine often used in e-liquid for tank-style vapes. This is considered to make disposable vapes easier to inhale for people who have never smoked [50]. ElfBar reusable pods are advertised as containing nicotine salts, and other pod based vapes have been found to include e-liquid with nicotine in this form [2,51]. Improving awareness of reusable pod-style vapes among young adults who use disposable vapes and intend to switch to smoking cigarettes when the disposable vape ban is implemented could protect these individuals from the harms of smoking by encouraging use of a less harmful alternative. However, it would be difficult to increase awareness and use specifically among this group without also increasing awareness and use among young people and those who might otherwise stop vaping without switching to smoking. Arguably, reusable pod-style vapes may be perceived as more of a commitment by youth, as we found they were by some young adults in this study, which could deter their use. Conversely, if they resemble disposables in appearance, cost, and availability, and if the pod lasts as long a singular disposable vape (as described by one of our participants), then both youth and young adults may treat them similarly, discarding the vape once the pod is empty. E-cigarette manufacturers often make new products or promote existing ones to evade restrictions [52–54], and popular brands have already launched pod vapes that are sold at a similar price with similar branding to disposable vapes [18]. It is possible that these or other new devices will replace disposable vapes now that the ban has been implemented (as disposables did after the FDA flavour ban in the US).

Two participants discussed sourcing disposable vapes after the ban, and participants discussed generally how the ban might encourage illegal sales of disposables. A qualitative study with young people in the UK (aged 16–20) conducted shortly before the enforcement of the ban reported that most believed they would be able to continue accessing disposable vapes after the restrictions [25]. If people continue to source disposable (or unregulated) vapes after the ban, this would pose a health risk as the vapes could contain banned, dangerous ingredients such as heavy metals or synthetic cannabinoids [55,56]. Recent testing of 510 confiscated vapes from 27 schools across regions of England found that 17.5% contained synthetic cannabinoids – a class of drug often referred to as ‘spice’ which have a considerably higher risk profile than cannabinoids typically found in cannabis [57]. Participants mentioned convenience shops as places where illegal sales currently occur, both underage sales and sales of currently banned products such as 50mg/ml vapes - a nicotine concentration well over the legal limit of 20mg/ml [16]. A study reported that, along with supermarkets, these shops have now overtaken vape shops as the most popular place to buy vape products [58]. If manufacturers can still export disposables to the UK as has been the case recently in Australia after a ban on disposable vapes [59], then sales may continue in some places in the UK, possibly in some convenience shops. Therefore, strong enforcement will be required to ensure the effectiveness of the ban. However, the industry has created similar products that meet the requirements of the law but can easily be treated like a disposable, so enforcement may not be required if people who vaped disposables simply swap to new legal products and use them as if they are disposables instead of purchasing illegal vapes*.* Future quantitative studies should explore the extent to which reusable vapes are being treated as disposables.

Although another study has explored opinions of impending vaping regulations in Australia among adolescents and adults (with limited exploration of banning disposables specifically) [60], this study is the first to our knowledge to assess the opinions and potential behavioural responses to the disposable vape ban in the UK among people who use disposable vapes. The qualitative nature of this research allowed us to gain more understanding and appreciation of the context and nuance of our data, revealing that most participants in our study who self-identified as having never smoked had tried cigarettes in the past. The young adult sample contributed to a rich exploration and detailed account of this demographic, and we reached information power for our sample as a whole. Our self-report measures resulted in the boundaries between our a priori categories being somewhat unclear, with many people who have ‘never smoked’ saying they had tried cigarettes in the past. Similarly, there is a growing number of people who vape and smoke cigarettes non-daily [61], like one of our participants in the previously smoking group who said they occasionally smoke cigarettes socially. There could be more participants in this group who also occasionally smoke. While it would be useful to investigate differences between groups, there was considerable within-group variation in smoking history, and this was not the aim of our study.

We did not reach information power for impacts of the ban specific to gender, as most of our participants were female. This was also not included in our research aims as we did not anticipate any differences between genders. We did not reach information power for adults over 22 either, as our participants were aged 18–22 except two who were aged 29 and 30. ASH reports that adults who vape aged 18–24 were the age group most likely to use disposable devices between 2022 and 2025 [32]. When this study was designed in 2023, use was particularly high among 18–24 year-olds (57% of those that vape) and 25–34 year-olds (47%), but use substantially dropped among 25–34 year-olds (to 28%) in 2024 when this study was conducted [32]. In addition, people who use disposables and have never smoked are more likely to be younger; Jackson and colleagues reported rare use of disposable vapes among adults who have never smoked (1.5%), but greater use among 18–24 year-olds who have never smoked (7.1%) [18]. Therefore, while our study may not capture the opinions and experiences of the full 18–30 age range of young adults who vape, it reflects the opinions and experiences of young adults within the age distribution who most used *disposable* vapes at the time of the study. Exact geographic location of participants was not collected, but it is highly likely the majority lived in Bristol and the surrounding area as recruitment primarily occurred via the University and posters around the campus and city. However, it is possible that some participants were residing outside this area as we also posted online adverts. The majority of participants were also white females, and roughly half were students at the University of Bristol. Vaping was reported as comparable between males and females in 2021, but in 2024, after the growth in popularity of disposables, a shift towards slightly greater use among females in 2024 was reported [18]. Although our predominantly female sample may be more representative of young adults who use disposable vapes, our sample still limits the generalisability of our findings to males, other ethnicities, those not at university, and adults aged 23 and older. The impacts of the ban for these other demographics may be different to those discussed by our participants. Future research should investigate the impacts of the ban on other age groups and with more diverse samples to ensure opinions and experiences are representative of the general public. Future work should also seek to objectively measure behavioural changes (e.g., smoking initiation, smoking frequency, alternative nicotine product use, or illicit product use) now that the ban has been implemented, but initial evidence seems to be supporting our findings, highlighting the usefulness of qualitative research in predicting potential responses to vaping regulations.

## Conclusions

This study provides insight into the potential consequences of banning disposable vapes in the UK for young adults. While many young adults may have switched to other vapes, some may use cigarettes as an alternative. Promoting awareness or encouraging the use of other vape products among adults needs to be balanced with preventing another product replacing disposable vapes among youths. In addition, access to disposable vapes may continue, possibly in convenience shops, where participants described current illegal sales. While the intentions and opinions of participants may change over time, the findings of this study could be useful to direct future research into the impacts of banning disposable vapes and could be used in conjunction with other evidence to mitigate unintended consequences of the ban.

## Supporting information

S1 Text Researcher positioning. (DOCX)

S2 Text Reasons for excluding participants from the analysis. (DOCX)

S3 Text Supplementary themes. (DOCX)

S1 Table Qualtrics survey and response options. (DOCX)

S2 Table Interview schedule for group N. (DOCX)

S3 Table Interview schedule for group D. (DOCX)

S4 Table Interview schedule for group P. (DOCX)
