# Supplementary material for: Exploring the potential consequences of the disposable vape ban in the UK: A qualitative study with young adults who use disposable vapes
Source: PLOS Glob Public Health. 2026 Mar 11;6(3):e0004686. doi: 10.1371/journal.pgph.0004686 (PMC12978755; doi:10.1371/journal.pgph.0004686)
Supplement: S1 Text — (DOCX) [file pgph.0004686.s001.docx]

**S1 Text. Researcher positioning**

The researcher (RA1) who analysed the data was a white 22-year-old male with a BSc (hons) in psychology from the University of Bristol. RA1 used to smoke cigarettes regularly and has occasionally used both refillable tank-style and disposable e-cigarettes. He was eager to investigate the use of disposable vapes among young adults having noticed an explosion in their popularity among his peers, including people who have never smoked regularly. At the time of data collection, RA1 was 21 and in his final year of the degree but data analysis occurred after the degree was obtained. RA1 has an interest in clinical psychology, particularly in drug use and harm reduction, and in reducing the societal stigma towards people who use drugs. He has experience supporting adults who use drugs and alcohol in Bristol and currently supports children with communication & interaction needs and moderate learning difficulties. RA1 had not met nor had any relationship with the participants before the study.
