# Supplementary material for: Exploring the potential consequences of the disposable vape ban in the UK: A qualitative study with young adults who use disposable vapes
Source: PLOS Glob Public Health. 2026 Mar 11;6(3):e0004686. doi: 10.1371/journal.pgph.0004686 (PMC12978755; doi:10.1371/journal.pgph.0004686)
Supplement: S2 Text — (DOCX) [file pgph.0004686.s002.docx]

**S2 Text. Reasons for excluding participants from the analysis**

N03: excluded because they stopped using disposable vapes one month before the interview.
N08: excluded because they switched to pod devices a year before the interview.
D03: excluded because they did not live in the United Kingdom.
D04: excluded because they did not live in the United Kingdom.
P06: excluded because they used the term ‘smoking’ to describe vaping, which created issues during the analysis.
