# Supplementary material for: Exploring the potential consequences of the disposable vape ban in the UK: A qualitative study with young adults who use disposable vapes
Source: PLOS Glob Public Health. 2026 Mar 11;6(3):e0004686. doi: 10.1371/journal.pgph.0004686 (PMC12978755; doi:10.1371/journal.pgph.0004686)
Supplement: S3 Text — (DOCX) [file pgph.0004686.s003.docx]

**S3 Text. Supplementary themes**

**Thoughts on cigarette and vape products**
The theme “thoughts on cigarette and vape products” includes two sub-themes: “comparison of smoking and vaping”, and “disposable vapes compared to reusable vapes”.
**Comparison of smoking and vaping**
 Some participants described vaping as more pleasant than smoking. When asked what they liked about disposable vapes, P02 said: *“tastes nicer than cigarettes [laughs] erm... yeah that’s it really.”* Similarly, N10 said: *“vaping yeah makes the taste of cigarettes a lot worse.”* Participants described off-putting elements of smoking. For example, D01 said: *“vapes you know taste really nice, erm the thing with cigarettes is yeah you end up covered in the smell of smoke in your fingers and your clothes.”* Participants also noted the difference between needing to go outside to smoke and being able to use vapes indoors. P02 said: “*you can have them (vapes) all the time, like you can just kind of vape in the house and stuff.”* Similarly, D02 said: *“you can hit it (vapes) anywhere [...] with cigarettes you have to make an event of it you have to go outside.”* Some also noted that the continuous use of vapes (compared to the end point of a cigarette) meant it is harder to monitor their nicotine intake. P01 said: *“sometimes I do like… just end up vaping for longer because I’m not really tracking the time.”*
**Disposable vapes compared to reusable vapes**
 Some participants said they preferred the taste of disposables to reusables. P03 said: *“the flavours (of disposables) are nicer.”* Similarly, P08 said*: “you know how like real ones start to burn and stuff, there’s all that kind of… issues (with reusables).”* Some participants also liked being able to switch between flavours easily with disposables. D02 said: *“that’s another appealing factor how quickly you can chop and change (flavours) when you’re getting sick of the other one.”* Although, N05 liked the consistency of reusables: “*if you’re someone like me who doesn’t really like to switch it up, you have one bottle, like… it’s just pretty constant.”*

Many participants described how they disliked the maintenance that was required with reusables, compared to the ease of disposables. D08 said: *“a lot of the disposable vapes, are fully charged until they die, erm when a normal erm refillable vape you will have to plug it in multiple times a day.”* Similarly, N01 said: *“it’s just so much more hassle like refilling it, and like cleaning the pods.”* When describing what they disliked about reusables, P08 said: *“if you don’t look after it, you just end up spending money trying to fix it, and that just seemed like an unnecessary thing.”*

**Reasons for vaping generally**
The theme “reasons for vaping generally” includes four sub-themes: “habit and addiction”, “ease of vaping”, “health reasons”, and “social influence”.
**Habit and addiction**
 Participants said that addiction was a reason they vaped. D01 said: *“it’s very much like a chemical addiction I would say.”* Similarly, P05 said: *“I’m definitely addicted to it now [...] I managed to quit for about three months but every single time I go through something stressful, I... like need one again.”* Other participants described vaping as a habit. When asked what were some of the reasons they vape, N02 said: *“just like habit really.”* Similarly, D06 said: *“I’m just used to it, nothing that really that it does to me, it’s just the habit of having a vape in my hand.”* A few participants said they felt they were not addicted to vaping. N07 said: *“I don’t feel like… I am addicted to it it’s just a… a habit at home [...] I don’t sort of miss it when I’m not here.”* Some participants noted that although they felt addicted to vaping, they also found it enjoyable. D01 said: *“they’re just kind of nice (disposable vapes) that’s like the bottom line, they’re just really nice.”* Similarly, P08 said: *“you know when you’re just on your computer it’s just a nice thing to kind of... do.”*

**Ease of vaping**
 When discussing why they vaped, participants often cited the ease of vaping. P04 said: *“if you want to smoke you have to go... outside I feel like you (can)... just constantly have a vape on you.”* Similarly, P08 said: *“vaping is a lot easier to do, under the radar [...] went from people having to step outside to smoke at a party, to just constantly having a nicotine... like, device in their hand.”* D02 said this meant they vape more than they smoke: *“the vape use is so much more frequent than smoking [..] I can hit it (vapes) anywhere, if I wanted to I could hit it in my room.”*

**Health reasons**
 Some participants said they vaped due for health reasons, as a less harmful alternative to cigarettes. D08 said: *“it first started as a replacement erm... for tobacco use, which was, I thought, was harming my health.”*  When asked why they switched from smoking to vaping, P07 said: *“Because I thought vapes are less bad than cigarettes. I don’t know if that’s true but in my head it was.”* Similarly, N07 said: “*it seems like a less harmful form of stress relief than… drinking or a different kind of behaviour.”* Like N07, a few other participants described how they tried to use vapes to improve their psychological health. When asked what their most important reason for vaping was, N02 said: *“the... de-stress, I find it quite calming, and like if I’m stressed... just... actually calm down.”* Similarly, P05 said vaping reduced their anxiety:

“*I could hardly go outside because my panic attacks were so bad, so I thought that if just having a vape, or like a cigarette or something, is going to make me go outside more then it’s… worth it, if that makes sense?”*

**Social influence**
 Many participants described how other people influenced their vape use. When asked what factors played a role in their decision to switch from smoking to vaping, P02 said: *“a lot of people around me, also... were vaping so that probably did play... quite a big role.”* Similarly, N06 said: *“I think the most important thing honestly is… like the social aspect of it it’s like we’re all sitting around having a chat, we’ll be vaping, we’ll like share each other’s.”* N09 said: *“I usually do it more when there’s loads of people around which is why I do it more on a night out.”* P05 described how they felt judged more when they were using cigarettes compared to vaping: *“this like family walked past me and this woman went oh my god can you smell that smoke, and I was like oh my gosh that is horrific, yeah.”*
