# Supplementary material for: Exploring the potential consequences of the disposable vape ban in the UK: A qualitative study with young adults who use disposable vapes
Source: PLOS Glob Public Health. 2026 Mar 11;6(3):e0004686. doi: 10.1371/journal.pgph.0004686 (PMC12978755; doi:10.1371/journal.pgph.0004686)
Supplement: S1 Table — (DOCX) [file pgph.0004686.s004.docx]

**S1 Table. Qualtrics survey questions and response options**

| **Survey Questions** | **Response options** |
| --- | --- |
| Please enter your participant ID number, ask the researcher if you are unsure what this is. | Open field box. |
| How old are you? (in years) | Open number field box. |
| What is your gender? | Male, Female, Transgender, Intersex, Non-binary, prefer not to say, I’d prefer to describe my gender in my own words (Open text box). |
| What is your ethnicity? | White, Mixed/multiple ethnicities, Asian/Asian British, Black/African/Caribbean/Black British, other ethnicity (open text box). |
| How long have you been using disposable vapes daily? | 3-6 months, 6-12 months, 1-2 years, 2-3 years, 3-4 years, 4-5 years, 5+ years. |
| Which brands of disposable vapes do you use? (e.g., Lost Mary, Elf Bar) | Open text box. |
| What nicotine strength/s do you use? (mg/ml or %). | Open text box. |
