# Supplementary material for: Exploring the potential consequences of the disposable vape ban in the UK: A qualitative study with young adults who use disposable vapes
Source: PLOS Glob Public Health. 2026 Mar 11;6(3):e0004686. doi: 10.1371/journal.pgph.0004686 (PMC12978755; doi:10.1371/journal.pgph.0004686)
Supplement: S3 Table — (DOCX) [file pgph.0004686.s006.docx]

**S3 Table. Interview schedule for dual-users**

| **Questions** | **Prompts** |
| --- | --- |
| Can you describe your vape use generally? | - How often do you vape? - Where do you usually vape? |
| What attracted you to disposable vapes initially? | Were there any specific reasons you chose to use disposable vapes?   - Can you describe the circumstances that led you to use disposable vapes for the first time? |
| What are some of the reasons you vape now? | - Of those reasons:   what’s the most important reason?   - Are there any other reasons? |
| What else do you like about disposable vapes? | - Could you elaborate on that? - Are there any other things you like about them? |
| Have you ever tried using a refillable vape? | - If yes:   What did you like or dislike about them? What makes you use disposable over refillable vapes?   - If no:  Have you considered using one? – ^Why/why not? |
| Have you ever tried to stop vaping? | - How? Did you use anything to help you? - How long did you stop for? - What was it like? - Have you tried to stop more than once? |
| Have you tried any other nicotine products before, even if it wasn’t to stop smoking or vaping: such as gum, lozenges, patches, snus, pouches? | - If they ever have: What was your experience of using them? - How do they compare to vaping? |
| If you had to spend a long time without vaping, like if you were on a long-haul flight, how would you feel? | - Imagine yourself in this situation and how it might feel. - Imagine not having your vape for eight hours. |
| If you went somewhere for the day and realised you forgot your vape and there was nowhere to buy one, what would you do? | - If disposable vapes weren’t available to buy for the day, what might you do? |
| The UK government are planning to ban disposable vapes, when this happens, what might you do? | - Try to imagine disposable vapes were no longer legal – what might you do? - Why? |
| Do you vape socially? If so, how will a ban affect this? | - How will a ban impact your vaping in pubs, at work, at university, when going out with friends? |
| How will a ban on disposable vapes affect your smoking patterns? | - Can you elaborate on that? |
| Has the government’s plan to ban disposable vapes affected your opinions on vaping? | - Why/why not? |
| How do you think a ban on disposable vapes will impact other people who vape but don’t smoke? | - How effective will a ban be to defer them from vaping? - How do you think it will impact younger people who can’t legally buy vapes? |
| Are there any comments you’d like to add? |  |
